# Global trends and burden of dental caries in permanent teeth (1990–2021): Epidemiological analysis, socio-demographic disparities, and future projections

**DOI:** 10.1097/MD.0000000000048438

**Published:** 2026-08-28

**Authors:** Lina Zhang, Siao Zhao

**Affiliations:** a Department of Stomatology, Nuclear Industry 215 Hospital of Shaanxi Province, Guiyang, P.R. China.

**Keywords:** ARIMA model, Global Burden of Disease, permanent caries, public health, socio‑demographic index

## Abstract

This study aims to analyze the incidence, prevalence, and years lost due to disability (YLDs) of permanent dental caries using Global Burden of Disease data, and to investigate its correlation with socioeconomic indicators. Data were obtained from the Global Burden of Disease (GBD) 2021 database, covering variations by age, sex, and geographic region. A retrospective epidemiological analysis was conducted using descriptive statistics, autoregressive integrated moving average (ARIMA) modeling, and correlation analyses between SDI and estimated annual percentage change (EAPC). Data visualization and statistical analyses were performed using R software. The global burden of dental caries has increased significantly, with a particular emphasis on low-SDI regions. The incidence rates of high-SDI countries were reduced as a consequence of enhanced access to dental care and fluoridation policies. The prevalence of caries among males increased modestly, with the maximum prevalence observed among those aged 15 to 24 years. A negative correlation was found between EAPC and SDI levels, indicating that the disease burden grew more rapidly in low-income regions. The rising global burden of dental caries, especially in low-SDI regions, highlights the urgent need for equitable oral health policies, enhanced access to dental care, and community-based preventive strategies to reduce global disparities.

## 1. Introduction

Dental caries in permanent teeth is 1 of the most prevalent oral diseases worldwide, characterized by the progressive destruction of dental hard tissues.^[1,2]^ As a chronic and irreversible condition, dental caries not only affects oral health but also has significant implications for overall health, quality of life, and socioeconomic development. The etiology of dental caries is multifactorial, involving dietary habits, oral hygiene practices, socioeconomic status, and regional development levels. Therefore, understanding the epidemiological patterns and disease burden of dental caries across different age groups, genders, geographic regions, and socio-demographic index (SDI) levels is crucial for formulating effective global prevention and control strategies.^[3]^

The Global Burden of Disease (GBD) study has been systematically assessing the incidence, prevalence, and Years Lived with Disability (YLDs) associated with various diseases since 1990.^[4]^ This comprehensive research provides valuable insights into global disease distribution and supports the evaluation of health policies across countries. In recent years, the burden and epidemiological trends of dental caries have exhibited notable changes due to socioeconomic development and improvements in living standards. In high-income regions, effective public health policies and oral health interventions have contributed to a decline in caries prevalence and disease burden. However, in low- and lower-middle-income regions, limited healthcare resources and a lack of oral health awareness have resulted in persistently high caries prevalence and increasing disease burden. Consequently, a deeper understanding of the epidemiological characteristics of dental caries across different SDI levels is essential for optimizing disease prevention strategies and resource allocation.^[5]^

Existing studies on the epidemiology of dental caries are often limited to specific countries or regions, lacking a comprehensive global perspective that considers diverse socioeconomic conditions and age groups. Furthermore, limited research has been conducted on the temporal trends of dental caries burden, particularly regarding future projections. This study utilizes the GBD database to systematically analyze the global and regional trends of dental caries from 1990 to 2021. It specifically examines variations in disease burden and incidence rates across different SDI levels, genders, and age groups while employing time-series forecasting models to estimate future trends over the next decade.

The primary objectives of this study are: To analyze the temporal trends of incidence, prevalence and YLDs rates of dental caries globally and regionally from 1990 to 2021. To explore the differences in epidemiological patterns of dental caries across SDI levels, genders, and age groups. To predict the future burden and incidence of dental caries up to 2050.

Through a systematic and multidimensional evaluation, this study aims to provide a comprehensive understanding of the global burden and future trajectory of dental caries. The findings will serve as a scientific foundation for the development of targeted prevention strategies, resource optimization, and policy formulation in global oral health management.

## 2. Methods

### 2.1. Data source and framework

Data for this study were obtained from the Global Burden of Disease (GBD) 2021 database (https://vizhub.healthdata.org/gbd-results/), which provides comprehensive epidemiological estimates for 371 diseases and injuries across 204 countries and territories. The database integrates information from national surveys, hospital registries, and epidemiological studies, ensuring high reliability and comparability. Data on dental caries in permanent teeth were extracted for the period 1990–2021, covering variations across socio-demographic index (SDI) levels, sexes, age groups, and geographic regions. The primary indicators analyzed included incidence, prevalence, and years lived with disability (YLDs), which were systematically modeled and age-standardized following GBD protocols.

### 2.2. Disease definition

According to GBD 2021 and the International Statistical Classification of Diseases, dental caries in permanent teeth is defined as the presence of a distinct cavity, undermined enamel, a softened floor or wall, a temporary or recurrent filling, or a tooth extracted due to caries. This standardized definition enables comparability across datasets and populations.

### 2.3. Measures of disease burden

Annual data on the incidence, prevalence, and YLDs of dental caries were collected from 1990 to 2021. The estimated annual percentage change (EAPC) was calculated to quantify temporal trends in age-standardized rates (ASRs). All rates were reported per 100,000 population with 95% uncertainty intervals (UIs), representing the 25th and 975th values from 1000 posterior distribution samples.

### 2.4. Statistical analysis and trend prediction

A retrospective epidemiological analysis was conducted using R software (version 4.4.3). Descriptive statistics were used to summarize incidence, prevalence, and YLDs across SDI regions, age groups, and sexes. The autoregressive integrated moving average (ARIMA) model was applied to assess temporal trends from 1990 to 2021 and to project incidence, prevalence, and YLDs through 2050. Model parameters (p, d, q) were optimized to ensure stationarity and minimize prediction error. Correlations between SDI and EAPC were examined using Pearson correlation analysis and linear regression, with statistical significance set at *P* < .05.

### 2.5. Geographical and visualization analysis

Geographic Information System (GIS)-based visualization was performed using the ggplot2, ggmap, and R ColorBrewer packages in R. Global distribution maps were generated to display spatial variations in the burden of dental caries in 2021. Additional visualizations included bar and line charts to illustrate temporal and demographic trends, scatter plots to depict EAPC–SDI relationships, and circular heatmaps to present longitudinal changes from 1990 to 2021.

### 2.6. Ethical statement

This study used publicly available data from the GBD 2021 database. As no individual-level or animal data were involved, ethical approval was not required. The study adhered to the Guidelines for Accurate and Transparent Health Estimates Reporting (GATHER).

## 3. Results

### 3.1. Global trends

This study analyzed the trends in untreated dental caries globally and regionally from 1990 to 2021. The results indicate that while the age-standardized burden of untreated dental caries has shown a slight decline over the past 30 years, the overall burden remains significantly high. This finding suggests that, despite advancements in oral health interventions, the improvement in the global caries burden has been limited.

For permanent teeth, the 2021 global incidence was estimated at 2370,414,032 cases (95% UI, 2099,098,156 to 2661,346,388) with an ASR of incidence 29,777.03 per 100,000 population (95% UI,26,310.27 to 33,490.91). The ASIR for permanent teeth since 1990 was 0.63% (95% UI, −10.40 to 13.01%), indicating a slight upward trend. The global prevalence of untreated periodontal disease in permanent teeth was estimated at 2242,874,758 cases (95% UI, 1957,579,677 to 2599,622,169) with an ASR of prevalence 27,543.34 per 100,000 population (95% UI, 23,976 to 32,018.09) and an ASPR of 0.52% (95% UI, −10.49% to 12.88%) (Table 1).

**Table 1:** An overview of the incidence, prevalence, and Years Lived with Disability (YLD) rates for permanent caries by gender globally and across various SDI regions in 2021, along with the corresponding age-standardized rates and estimated annual percentage change (EAPC) values.

Area	2021		2021		2021		EAPC 1990–2021		
	Incidence	ASR (per 100,000)	Prevalence	ASR (per 100,000)	YLDs	ASR (per 100,000)	ASIR	ASPR	ASYR
Both (Global)	2,370,414,031.84 (2,099,098,156.09–2661346387.71)	29,777.03 (26,310.27–33490.91)	2,242,874,758.25 (1,957,579,677.47–2599622169.36)	27,543.34 (23976–32018.09)	2,198,064.47 (988183–4174724.95)	27.02 (12.1–51.36)	0.63% (−10.40% to 13.01%)	0.52% (−10.49% to 12.88%)	0.52% (−10.49% to 12.88%)
Female (Global)	1,179,064,868.04 (1,045,956,815.57–1324945905.91)	29,877.72 (26,437.48–33602.38)	1,131,593,062.5 (987,306,930.52–1307857024.05)	27,791.06 (24,168.74–32248.26)	1,102,863.57 (497,295.6–2084214.29)	27.14 (12.15–51.39)	0.62% (−9.75% to 12.18%)	0.52% (−9.83% to 12.06%)	0.51% (−9.84% to 12.05%)
Male (Global)	1,191,349,163.8 (1,052,116,661.34–1337867523.9)	29,678.33 (26,169.01–33359.6)	1,111,281,695.75 (969,787,614.02–1294372240.55)	27,300.85 (23,802.34–31795.78)	1,095,200.89 (490,401.87–2090510.66)	26.91 (12.02–51.33)	0.63% (−9.75% to 12.21%)	0.52% (−9.84% to 12.07%)	0.52% (−9.84% to 12.07%)
Low SDI	330,220,061.3 (288,016,584.13–375331728.97)	29,403.82 (25,801.98–33007.58)	310,714,532.51 (267,852,988.3–360694837.76)	31,719.14 (27,960.28–36261.48)	305,981.26 (133,494.2–574356.99)	30.97 (14.01–57.73)	−0.02% (−0.05% to 0.01%)	−0.10% (−0.16% to −0.04%)	−0.08% (−0.15% to −0.02%)
Low-middle SDI	623,984,153.92 (545,108,731.13–703956895.32)	31,182.47 (27,260.31–35209.32)	566,930,050.64 (488,469,072.45–668027834.77)	29,891.17 (25,921.28–34837.22)	555,886.81 (246,485.65–1059748.1)	29.18 (13.04–55.36)	−0.03% (−0.09% to 0.04%)	−0.06% (−0.18% to 0.07%)	−0.05% (−0.17% to 0.07%)
Middle SDI	747,613,546.44 (661,350,067.11–845051324.07)	29,951.73 (26,314.08–33821.61)	704,937,397.53 (607,791,504.82–824675289.26)	27,257.52 (23565–31966.2)	691,531.67 (311,276.68–1329672.65)	26.77 (11.97–51.3)	0.51% (0.44% to 0.57%)	0.06% (−0.01% to 0.14%)	0.06% (−0.01% to 0.14%)
High-middle SDI	354,901,014.01 (312,769,940.92–402702371.86)	28,068.89 (24,352.04–31973.91)	379,284,830.04 (328,955,920.38–436467219.13)	26,691.98 (23,040.41–31381.8)	371,028.93 (167,789.85–710272.62)	26.27 (11.65–49.92)	0.39% (0.29% to 0.48%)	−0.20% (−0.21% to −0.18%)	−0.19% (−0.21% to −0.18%)
High SDI	311,697,352.44 (280,824,482.65–345208230.36)	30,049.02 (26,769.17–33354.51)	278,799,405.2 (248,570,842.18–314093138.37)	23,472.19 (20,786.3–26991.46)	271,470.99 (124,442.9–518668.26)	23.06 (10.27–43.91)	0.02% (−0.02% to 0.07%)	−0.22% (−0.27% to −0.17%)	−0.22% (−0.27% to −0.17%)

Values in parentheses represent 95% uncertainty intervals.

ASIR = age-standardized incidence rate, ASPR = age-standardized prevalence rate, ASR = age-standardized rate (per 100,000 population), ASYR = age-standardized years lived with disability (YLD) rate, EAPC = estimated annual percentage change, SDI = socio-demographic index.

Table 1 demonstrates that although age-standardized rates (ASRs) remained relatively stable over the past 3 decades, the absolute number of cases increased substantially. In addition, the Estimated Annual Percentage Change (EAPC) values show heterogeneous trends across SDI regions. High-SDI regions exhibited declining ASPR and ASYR, whereas middle and high-middle SDI regions showed modest increases in incidence (ASIR), suggesting that socioeconomic development alone does not fully offset behavioral and environmental risk factors. Notably, low and low-middle SDI regions maintained high ASRs despite slight declines in EAPC, indicating a persistently heavy baseline burden. These findings highlight the structural inequality underlying global caries distribution.

### 3.2. Age, gender, and socioeconomic differences

For permanent caries, the incidence rate increases with age, starting from age 5 and peaking between ages 20 and 24, after which it gradually decreases. Prevalence and YLD rates for permanent caries exhibit a similar pattern, increasing after age 5, peaking at ages 20–24, and then gradually declining.

In 2021, there was a minimal difference in the prevalence of permanent caries between males and females globally, indicating that gender may not be a significant factor in the incidence of untreated dental caries.

**Figure 1.**
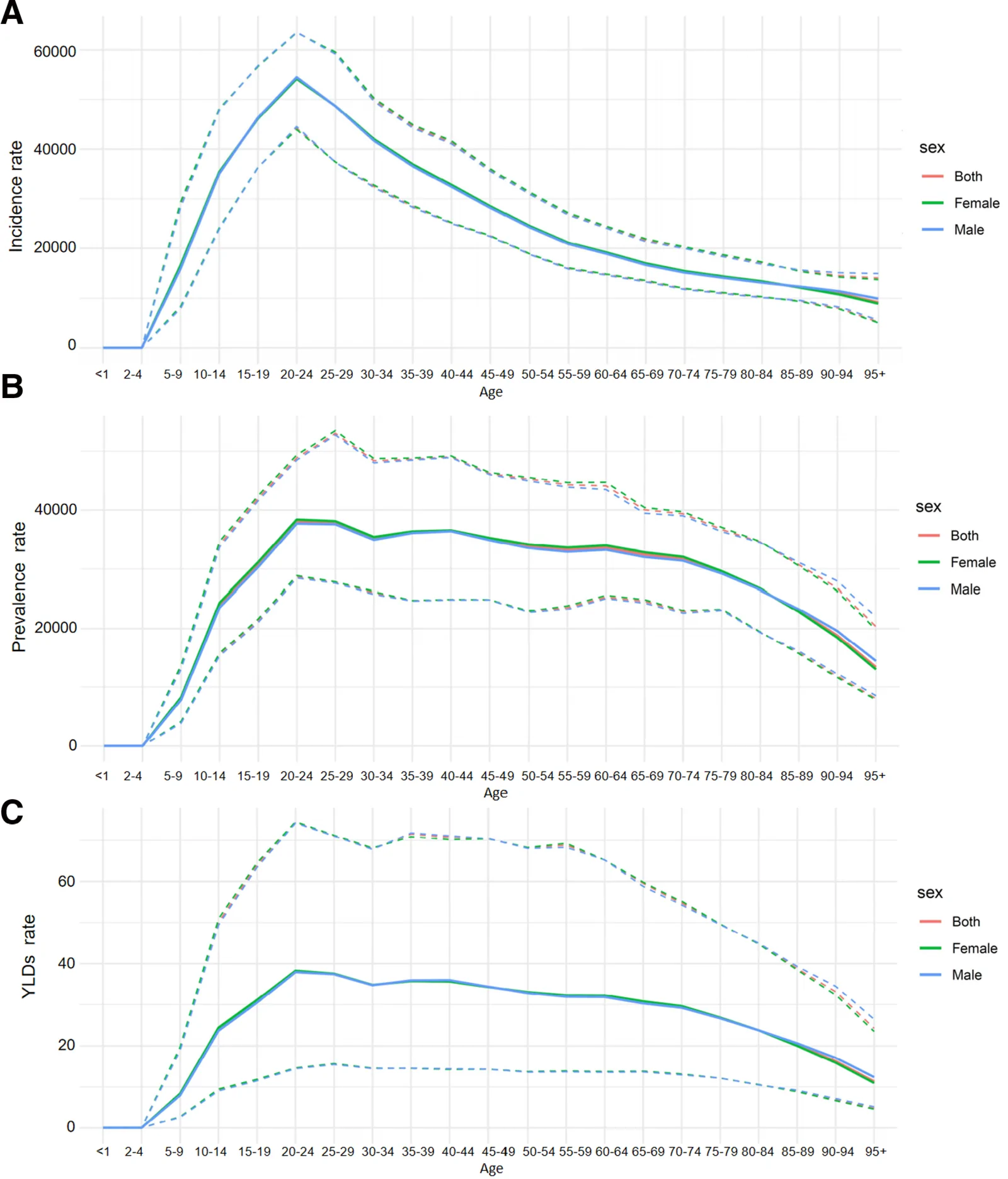
The global disease burden of permanent caries across different age groups and genders in 2021. The dashed lines represent the 95% uncertainty intervals (UI). (A) Incidence rate, (B) Prevalence rate, (C) YLD rate (per 100,000 population).

For permanent caries, as indicated by the fitted trend lines, East Asia has the lowest age-standardized incidence rate, while Tropical Latin America has the highest. There appears to be no strong correlation between SDI values and age-standardized incidence rates for permanent caries. However, for ASPR and ASYR, similar trends emerge, with High-income Asia Pacific showing the lowest rates and Andean Latin America and Southern Latin America having the highest rates. Overall, regions with high SDI values display relatively lower age-standardized prevalence and YLD rates, whereas regions with lower SDI values exhibit higher rates. Annual changes between 1990 and 2021 show considerable fluctuation in age-standardized prevalence and YLD rates across most regions, with a general downward trend (Fig. 1).

### 3.3. Geographic variations

The lowest ASR of incidence for permanent caries was observed in Taiwan (Province of China) at 13,329.27 per 100,000 population (95% UI, 10,258.63 to 16,272.28), followed by the Democratic People’s Republic of Korea at 14,287.89 (95% UI, 10,984.1 to 17,839.22) and Finland at 22,482.22 (95% UI, 18,513.59 to 27,495.03). On the other hand, Cambodia reported the highest for permanent teeth at 37,688.44 per 100,000 population (95% UI, 32,668.05 to 42,766.89), with Indonesia (36,825.74; 95% UI, 32,315.23 to 41,419.18) and Brazil (36,800.64; 95% UI, 32,044.07 to 41,659.97) also ranking among the highest.

**Figure 2.**
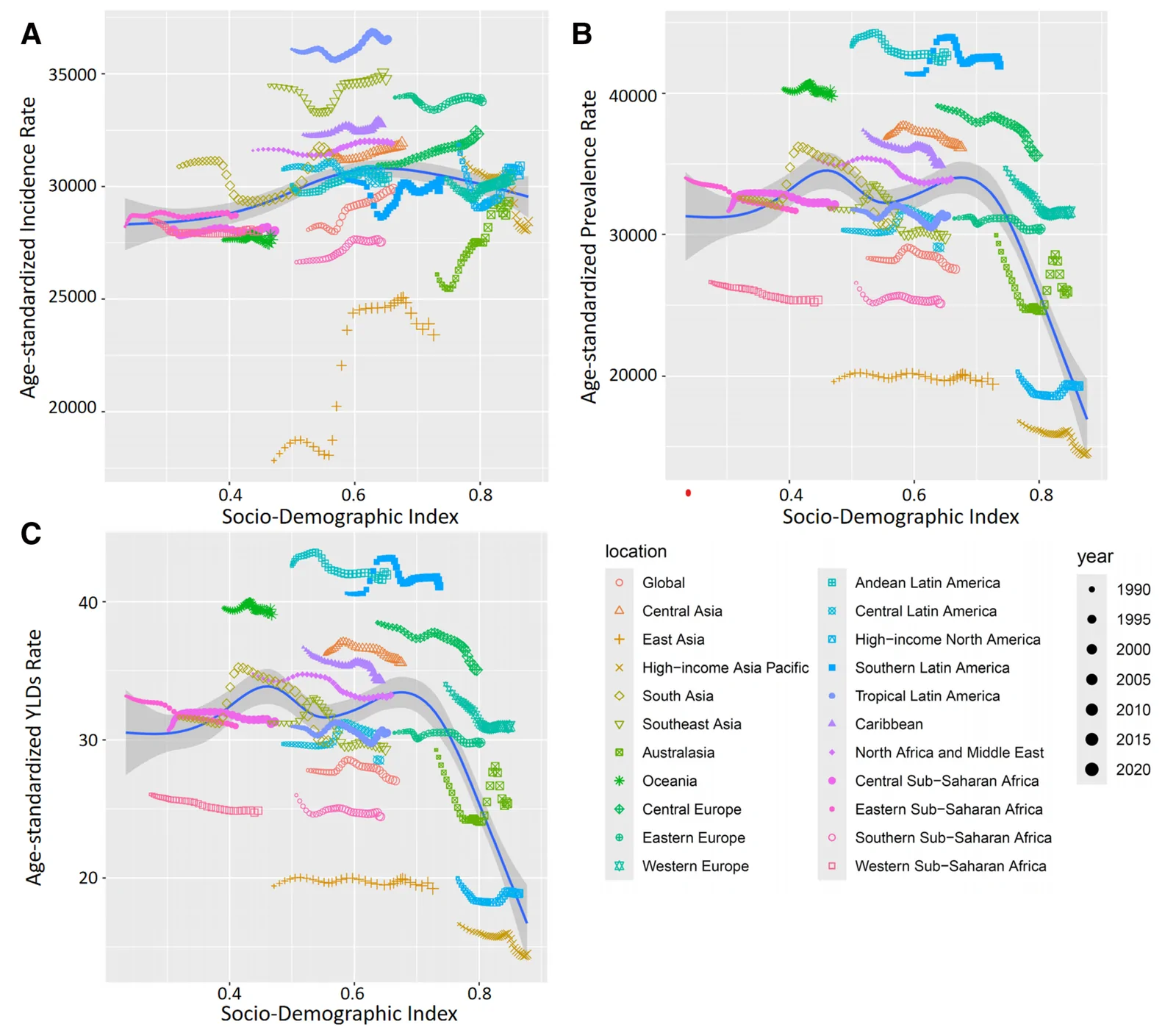
The relationship between age-standardized rates of disease burden for permanent caries and SDI values across different regions from 1990 to 2021. (A) Incidence rate, (B) Prevalence rate, (C) YLD rate (per 100,000 population).

For permanent caries, high-middle SDI regions recorded the lowest ASR of incidence at 28,068.89 per 100,000 population (95% UI, 24,352.04 to 31,973.91), while low-middle SDI regions had the highest at 31,182.47 (95% UI, 27,260.31 to 35,209.32). Similarly, ASR rates of prevalence and YLD were lowest in high-SDI regions and highest in low-SDI regions. From 1990 to 2021, ASIR showed a decreasing trend in low and low-middle SDI regions but an increasing trend in other regions. ASPR and ASYR demonstrated an increasing trend in middle-SDI regions, while other regions experienced a decline (Fig. 2).

### 3.4. Future trend projections

Using the ARIMA model, we projected the incidence, prevalence, and YLD rates for permanent caries worldwide and across various SDI regions over the next 29 years (2022–2050).

**Figure 3.**
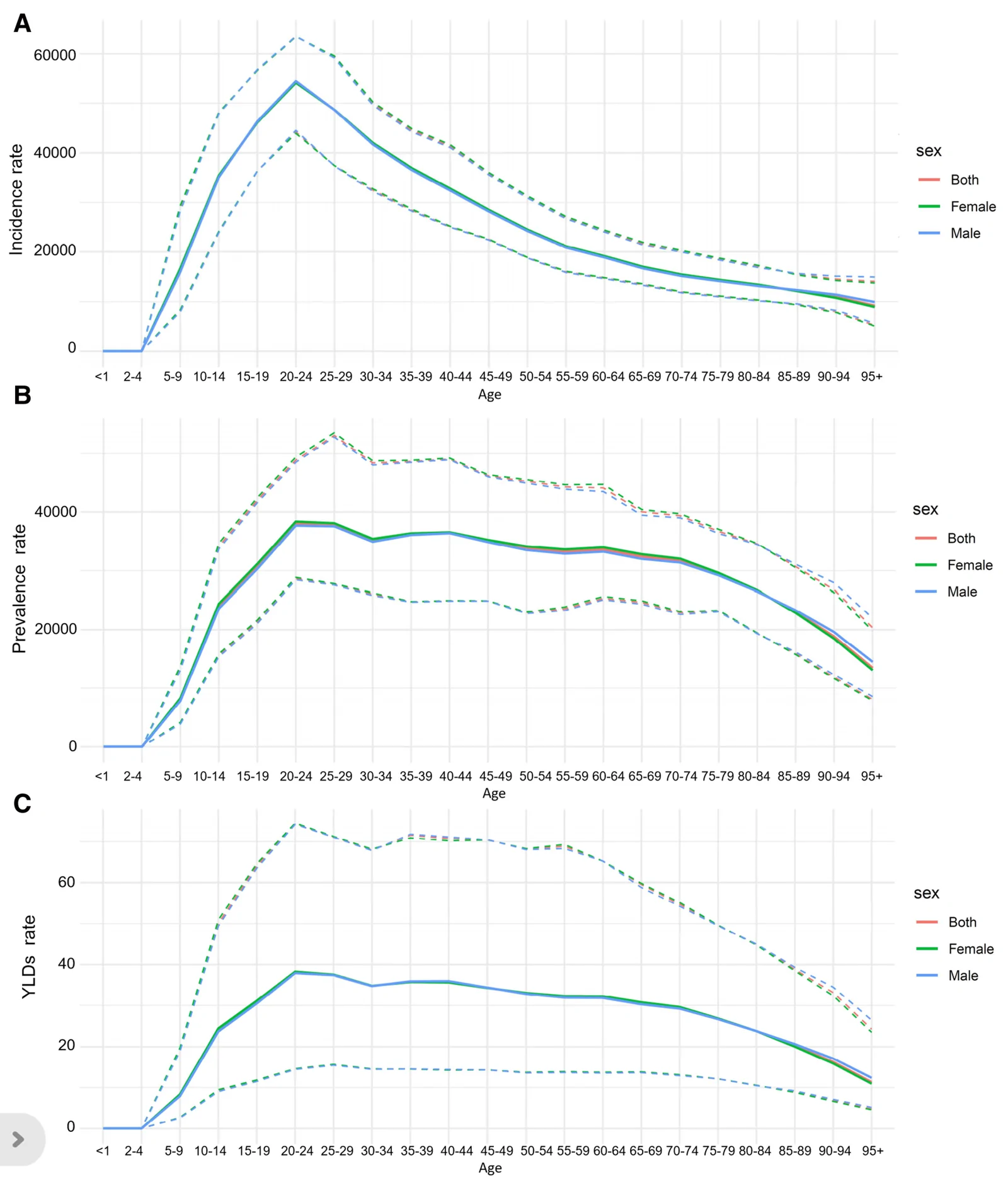
The trends and forecasts of age-standardized rates of disease burden for permanent caries across different SDI regions from 1990 to 2050. (A) Incidence rate, (B) Prevalence rate, (C) YLD rate (per 100,000 population).

For permanent caries, ASIR showed considerable fluctuation between 1990 and 2021. The high-middle and middle-SDI regions displayed an overall upward trend, while other regions experienced a slight decrease or relatively stable trends. Notably, the ASIR in high-SDI regions remained consistently below the global average. The ASPR and ASYR of permanent caries followed similar trends with minimal overall changes, with the burden in high-SDI regions staying below the global level throughout the period (Fig. 3).

Due to significant fluctuations in ASIR for permanent caries from 1990 to 2021, the 2022–2050 forecast curves based on the ARIMA model also exhibit substantial variability, resulting in wider confidence intervals. Except for the low-SDI region, other areas are anticipated to have minimal changes in ASPR and ASYR in the coming years.

## 4. Discussion

We examined trends in dental caries of permanent teeth from 1990 to 2021 across SDI strata, age, sex, and region. Across the 3 decades, incidence, prevalence, and YLDs rose worldwide, with the steepest increases in low- and low-middle-SDI settings. The picture is not uniform: several high-SDI countries now show flatter trajectories or modest declines, whereas many resource-constrained regions continue to accumulate untreated disease and disability.^[6]^

Several mechanisms plausibly sit behind these patterns. Demography matters: a larger global population and longer life expectancy expand the pool at risk and extend exposure time. Diet has also shifted. Urbanization and the spread of inexpensive, sugar-dense foods and beverages have altered everyday intake in many countries; the effect is particularly visible where processed products are cheap and fresh alternatives are not. At the same time, access to prevention and basic care remains uneven. Regular fluoride use, routine checkups, and timely restorative treatment are commonplace in high-income health systems but are sporadic or unaffordable for many families in lower-SDI settings.^[7]^ Reporting and coding differences may contribute to between-country contrasts, yet they are unlikely to account for the consistent gradients observed.^[8,9]^

The SDI gradient is clearest when prevention policies are considered. Where fluoridated toothpaste is widely used, water fluoridation or comparable population-level measures exist, and school-age care is routine, caries levels tend to be lower and stable.^[10]^ In contrast, low- and low-middle-SDI regions commonly face thin workforces, limited public financing for oral health, and household out-of-pocket barriers. In such environments, even modest gaps – no fluoride toothpaste at home, no community prevention, delayed visits – compound over time into higher prevalence and more years lived with disability.^[6,11]^

Age and sex patterns add nuance. Burden peaked at 15 to 24 years, a period marked by frequent snacking, sugary drinks, and inconsistent hygiene, while older adults showed lower prevalence, likely reflecting cumulative tooth loss and fewer at-risk tooth surfaces. Males carried slightly higher incidence than females in most strata, although differences were small. Behavioral explanations are credible – men tend to brush less consistently and attend fewer preventive visits – yet the gap was neither large nor universal, suggesting local culture and service organization also play a role.^[12]^

Geography still matters. In 2021, Tropical Latin America, Andean Latin America, and Southern Latin America ranked among the highest-burden regions, whereas Australasia, Western Europe, and East Asia were lowest. Economic development and long-standing prevention programs help explain the latter group’s position; constrained clinical capacity and high household costs help explain the former.^[13]^ Dietary patterns and social norms around oral health – how early children start brushing with fluoride, whether families seek care when asymptomatic – likely amplify these structural factors.

Our trend metrics offer one more perspective. The negative correlation between SDI and the EAPC for YLDs suggests that better-resourced systems are not only preventing disease but also limiting disability when disease occurs. By contrast, the weaker relationship between SDI and the EAPC for incidence implies that behaviors, local food environments, and policy choices can push incidence up or down irrespective of national income alone. In other words, resources help, but they do not substitute for effective prevention.

Forecasts to 2050 point in the same direction: continued growth in prevalence and YLDs in lower-SDI regions unless policy changes. Practical responses are well-known but unevenly implemented – reliable access to fluoride (toothpaste as a baseline; community fluoridation where feasible), early and regular prevention in schools, brief dietary counseling integrated into primary care, and timely, affordable basic treatment. Investments in frontline workforces and simple procurement policies (e.g., ensuring low-cost fluoride toothpaste is available and taxed appropriately) can deliver outsized returns. The political economy of water fluoridation and the costs of scaling preventive services are real constraints; where fluoridation is not feasible, alternatives such as targeted varnish programs and school-based interventions should be prioritized.

This analysis has limitations. Estimates from low-SDI settings may understate true burden because surveillance systems are incomplete and care is often delayed or absent. Diagnostic thresholds and reporting practices differ across countries and over time, which can affect comparability. Finally, we relied on aggregate data; individual-level behaviors, diets, and contextual influences were not modeled and could clarify causal pathways in future work.

Future research should further investigate the determinants underlying the observed SDI-related disparities. Specifically, multilevel analyses incorporating sugar consumption patterns, fluoride exposure, oral health workforce density, health expenditure, urbanization rate, and education level may help clarify causal pathways. Longitudinal cohort studies and policy evaluation studies could also assess the effectiveness of community-based fluoridation programs, school-based prevention initiatives, and primary oral healthcare integration. Such analyses would provide more actionable evidence for targeted interventions in high-burden regions.

Taken together, the global burden of permanent-teeth caries has grown over 3 decades, with sharp disparities by region and development level and a distinct peak in late adolescence and early adulthood. The tools to bend these curves – fluoride, basic prevention, timely treatment – are simple and cost-effective. Making them reliably available, especially in lower-SDI settings, is the hard part. Progress will depend on steady investment in primary oral healthcare, policy choices that favor prevention, and culturally attuned programs that make healthy behaviors easier to choose.

## 5. Conclusion

This study demonstrates that although age-standardized rates of permanent dental caries have remained relatively stable globally, the absolute burden has increased markedly, with the most pronounced growth occurring in low- and low-middle SDI regions. The peak burden observed among adolescents and young adults (15–24 years) highlights a critical window for preventive intervention.

From a clinical perspective, these findings emphasize the need for earlier preventive engagement, including routine fluoride exposure, dietary counseling, fissure sealant programs, and strengthened school-based oral health services. Clinicians in high-burden regions should prioritize early detection and minimally invasive management to reduce long-term disability. At the policy level, improving equitable access to basic preventive care and affordable treatment in low-SDI regions is essential to curb widening global disparities.

If current trends persist, projections to 2050 suggest continued pressure on oral healthcare systems, particularly in resource-limited settings. Therefore, integrating oral health into universal health coverage frameworks and reinforcing population-level prevention strategies should be considered urgent global health priorities.

## Acknowledgments

The Authors thank all the individuals who contributed to the Global Burden of Disease Study 2021 for their extensive support in finding, cataloguing, and analyzing data and facilitating communication between and among team members.

## Author contributions

**Data curation:** Lina Zhang.

**Investigation:** Lina Zhang.

**Methodology:** Lina Zhang.

**Supervision:** Siao zhao.

**Writing – original draft:** Lina Zhang.

**Writing – review & editing:** Siao Zhao.
